# Single cell sequencing revealed the mechanism of CRYAB in glioma and its diagnostic and prognostic value

**DOI:** 10.3389/fimmu.2023.1336187

**Published:** 2024-01-11

**Authors:** Hua-Bao Cai, Meng-Yu Zhao, Xin-Han Li, Yu-Qing Li, Tian-Hang Yu, Cun-Zhi Wang, Li-Na Wang, Wan-Yan Xu, Bo Liang, Yong-Ping Cai, Fang Zhang, Wen-Ming Hong

**Affiliations:** ^1^ Department of Neurosurgery, First Affiliated Hospital of Anhui Medical University, Hefei, China; ^2^ Shandong University of Traditional Chinese Medicine, Jinan, Shandong, China; ^3^ Department of Pathology, School of Basic Medical Sciences, Anhui Medical University, Hefei, China; ^4^ Department of Pathology, Anhui Medical University, Hefei, Anhui, China; ^5^ School of Nursing, Anhui Medical University, Hefei, China; ^6^ Department of Dermatology and Venereology, First Affiliated Hospital of Anhui Medical University, Hefei, China; ^7^ Open Project of Key Laboratory of Dermatology, Ministry of Education, Anhui Medical University, Hefei, China

**Keywords:** glioma, CRYAB, diagnosis, prognosis, immune infiltration, tumor immune microenvironment

## Abstract

**Background:**

We explored the characteristics of single-cell differentiation data in glioblastoma and established prognostic markers based on CRYAB to predict the prognosis of glioblastoma patients. Aberrant expression of CRYAB is associated with invasive behavior in various tumors, including glioblastoma. However, the specific role and mechanisms of CRYAB in glioblastoma are still unclear.

**Methods:**

We assessed RNA-seq and microarray data from TCGA and GEO databases, combined with scRNA-seq data on glioma patients from GEO. Utilizing the Seurat R package, we identified distinct survival-related gene clusters in the scRNA-seq data. Prognostic pivotal genes were discovered through single-factor Cox analysis, and a prognostic model was established using LASSO and stepwise regression algorithms. Moreover, we investigated the predictive potential of these genes in the immune microenvironment and their applicability in immunotherapy. Finally, *in vitro* experiments confirmed the functional significance of the high-risk gene CRYAB.

**Results:**

By analyzing the ScRNA-seq data, we identified 28 cell clusters representing seven cell types. After dimensionality reduction and clustering analysis, we obtained four subpopulations within the oligodendrocyte lineage based on their differentiation trajectory. Using CRYAB as a marker gene for the terminal-stage subpopulation, we found that its expression was associated with poor prognosis. *In vitro* experiments demonstrated that knocking out CRYAB in U87 and LN229 cells reduced cell viability, proliferation, and invasiveness.

**Conclusion:**

The risk model based on CRYAB holds promise in accurately predicting glioblastoma. A comprehensive study of the specific mechanisms of CRYAB in glioblastoma would contribute to understanding its response to immunotherapy. Targeting the CRYAB gene may be beneficial for glioblastoma patients.

## 1 Introduction

Gliomas present a prominent hazard due to their prevalent occurrence as the predominant neoplasms originating in the adult brain (1). Diagnosing and treating the majority of neurological disorders can be inherently challenging due to their complex etiology, difficulties in studying the underlying pathophysiological mechanisms, and the limited progress in the development of corresponding medications or therapies. As of now, the therapeutic options for patients with gliomas, in particular, remain considerably limited. The immune suppressive effects of gliomas present significant challenges for immunotherapy, while conventional approaches such as surgery, radiation, and chemotherapy have shown minimal efficacy (2). Consequently, early diagnosis, precise prognosis, and intervention is essential (3). Over the years, the early diagnosis and effective intervention of gliomas have faced substantial challenges. Although significant and encouraging progress has been made in exploring the heterogeneity between and within tumors, leveraging this heterogeneity for diagnosis and investigating the tumor microenvironment has further advanced immunotherapy (4). Nevertheless, glioma patients have benefited minimally from these developments (5). Therefore, urgent research efforts are required to identify novel and more suitable approaches to combat gliomas (6). Simultaneously, it is imperative to acquire a comprehensive comprehension of the underlying pathogenic mechanisms and prospective targets linked to the commencement and advancement of gliomas in order to facilitate the exploration of groundbreaking advancements (7).

The αB-crystallin protein, encoded by the CRYAB gene, belongs to the family of heat shock proteins. It is predominantly found in muscles and the nervous system, and it functions as a molecular chaperone and antioxidant under stress conditions (8). Mutations or abnormal expression of the CRYAB gene are associated with various diseases (9). In the nervous system, CRYAB gene mutations can lead to familial progressive spinal muscular atrophy (FPSMA), which is an inherited neurodegenerative disease (10). FPSMA primarily affects motor neurons, resulting in progressive muscle weakness and atrophy (11). αB-crystallin is a critical factor in the maintenance of neuronal functionality, and alterations in the CRYAB gene can result in aberrant αB-crystallin production. This, in turn, impairs normal neuronal function and survival, ultimately contributing to the manifestation of symptoms associated with FPSMA (12). Moreover, it has been observed that the CRYAB gene also exerts an influence on other neurodegenerative disorders, including Alzheimer’s disease and Parkinson’s disease (13, 14). Abnormal expression of the CRYAB gene in tumors has been extensively studied and is associated with the biological characteristics and prognosis of cancers (10). In certain types of cancers, including breast cancer (15), prostate cancer (16), and glioma (17), overexpression of the CRYAB gene is associated with malignancy. Overexpressed αB-crystallin promotes the survival and proliferation of tumor cells by inhibiting apoptosis processes and regulating proteins involved in the cell cycle (18). It also has an impact on cell migration and invasion, as high expression of CRYAB is correlated with enhanced metastatic and invasive capabilities of tumor cells. Moreover, CRYAB may alter the morphology and migration ability of tumor cells by promoting epithelial-mesenchymal transition (EMT) and activating other related signaling pathways (19). Furthermore, overexpression of CRYAB may contribute to increased resistance of tumor cells to chemotherapy drugs. It can enhance tumor cell survival and decrease sensitivity to treatment through various mechanisms, such as alleviating cellular stress and regulating intracellular calcium ion concentration. Hence, the CRYAB gene assumes a crucial role in both the development of FPSMA within the nervous system and the initiation and advancement of tumor formation. Conducting comprehensive investigations into the molecular mechanisms and functionalities of CRYAB will greatly contribute to enhancing our comprehension of the pathogenesis of associated disorders.

Existing literature has reported that the expression level of the CRYAB gene is usually elevated in glioblastomas (20). Glioblastoma is a highly invasive malignant brain tumor, characterized by significant biological heterogeneity and varying treatment responses. Abnormal expression of the CRYAB gene has become a focus in studying the mechanisms underlying glioblastoma development (21). In the realm of cellular stress response, CRYAB, an integral member of the heat shock protein (HSP) family, actively engages in cellular stress responses, affording protection to cells against the deleterious effects provoked by environmental stressors and cellular stressors. In glioblastoma, the high expression of CRYAB may help tumor cells adapt to adverse environments and alleviate cellular stress, thereby promoting their survival and proliferation. Regarding cell cycle regulation, CRYAB has also been found to regulate the cell cycle of glioblastoma cells. The upregulation of CRYAB is linked to abnormal expression of cell cycle-associated proteins and disturbances in the cell cycle. This may lead to excessive cell proliferation and rapid tumor development. Tumor cells often suppress apoptosis and exhibit excessive proliferation. The overexpression of CRYAB may inhibit apoptosis in glioblastoma cells, thereby increasing their survival and resistance to treatment. This may be related to the regulation of apoptosis-related proteins (such as the Bcl-2 family) and the antioxidant system by CRYAB in the process of cell apoptosis. Although the specific functions and interaction mechanisms of CRYAB in glioblastoma are not fully understood, it is considered to be one of the important factors involved in glioblastoma occurrence and development (22). Further comprehensive investigations are pivotal for determining the precise involvement of CRYAB in glioblastoma and may yield promising therapeutic targets for novel treatment approaches.

Researchers in the field of glioblastoma immunology are strongly committed to unraveling the intricate interplay between the immune system and the pathogenesis of glioblastoma, as well as harnessing immunotherapeutic interventions to potentiate the immune-mediated antitumor response against glioblastoma (23). Glioblastoma is a highly heterogeneous and immunosuppressive malignant tumor (24). One of the goals of immunological research is to uncover how glioblastoma inhibits the immune system’s attack and evades immune surveillance (25). In investigations concerning immune cell infiltration, advanced methods like single-cell sequencing are employed to scrutinize the distribution and abundance of immune cells in the tumor microenvironment. This allows for a comprehensive assessment of the immune cell infiltration surrounding the tumor (26). This helps to understand the types, quantities, and activities of immune cells in glioblastoma. In addition, signaling pathways that regulate immune responses, known as immune checkpoints, are studied in glioblastoma. As an example, researchers delve into analyzing the activation and inhibition of crucial signaling pathways like PD-1/PD-L1 and CTLA-4 (27). Understanding the impact of immune checkpoint signaling on immune cell function may contribute to the development of immunotherapies targeting these signaling pathways (28). In recent years, emerging immunotherapy approaches involve the use of CAR-T cell therapy or T-cell receptor (TCR) gene-engineered T cells to target and attack tumor cells (29). These engineered immune cells can enhance the anti-tumor effects by targeting tumor-specific antigens. Furthermore, researchers are also focused on developing vaccines to stimulate the immune system’s response against glioblastoma. These vaccines can include tumor-associated antigens (TAAs) or neoantigens to stimulate a specific immune response against tumor cells (30). The objective of these research efforts is to find new strategies to overcome immune tolerance and resistance in glioblastoma, improve patients’ response to immunotherapy, and enhance treatment outcomes. The progress in immunological research brings hope to glioblastoma patients by providing more effective treatment options.

Historically, research on neurological disorders has predominantly focused on histology, physiology, and sequencing at the cellular population level. However, this approach faces constraints when performing transcriptomic analysis at the single-cell level, hindering the precise identification of pathological alterations and immunological characteristics associated with these diseases. In this study, we conducted snRNA-seq on tumor samples from 10 GBM patients, companying utilizes scRNA-seq and transcriptomic data. Single-cell sequencing technology enables the determination of the gene expression profiles of individual cells, thus allowing the comparison of variations in the quantity of specific cell subpopulations and transcriptomic alterations within each subpopulation. Through dimensionality reduction and clustering, we identified four subpopulations within the glioblastoma (GBM) tumor microenvironment of oligodendrocyte-like cells. We further investigated the differentiation relationships between these subpopulations and explored the origin of GBM cells. By unraveling the intricate mechanisms driving the onset and progression of GBM, this study contributes groundbreaking perspectives that hold great promise for personalized therapeutic interventions, ultimately leading to enhanced prognostic outcomes for individuals affected by GBM.

## 2 Methods

### 2.1 Data source

The SnRNA-seq data utilized in this study were retrieved from the Gene Expression Omnibus (GEO) repository, which is hosted by the National Center for Biotechnology Information (NCBI). Additional information and access to the repository can be found at the following URL: https://www.ncbi.nlm.nih.gov/geo/. The specific dataset used in this study is identified by the GSE number GSE138794. The samples analyzed include GSM4119521, GSM4119522, GSM4119523, GSM4119524, GSM4119525, GSM4119526, GSM4119527, GSM4119528, GSM4119529, GSM4119530. Furthermore, bulk RNA-seq data were obtained from the official website of The Cancer Genome Atlas (TCGA), accessible at https://portal.gdc.cancer.gov/.

### 2.2 Data filtering and the standard process

The unprocessed snRNA-seq data was acquired and transformed into a count matrix. Subsequently, snRNA-seq data analysis was conducted using the Seurat package (version 4.3.0) implemented in the R programming language (version 4.2.0), following the methodologies outlined in previous scientific investigations (31, 32). To filter out low-quality cells and potential doublets, we utilized the DoubletFinder package (version 2.0.3) with its standard workflow (33). Quality control criteria were applied to retain cells for further analysis. Only cells that met the following criteria were included: 300 < nFeature < 7,500, 500 < nCount < 100,000, mitochondrial gene expression accounting for less than 20% of the total expressed genes per cell, and erythroid gene expression accounting for less than 5% of the total expressed genes per cell. To mitigate variations in library size and cell-specific biases, the count matrix underwent normalization. Highly variable genes are identified based on their expression variance across cells. Normalization was performed after identifying the top 2000 highly variable genes. We employed the Harmony R package (version 0.1.1) to alleviate batch effects observed within the samples, as previously reported in relevant literature (34, 35). Further downscaling and clustering analysis were conducted using the top 30 principal components (PCs). The UMAP method was employed to visualize the cellular heterogeneity in a two-dimensional (2D) map. To annotate the cell clusters, we referred to known cell markers from previous literature and the CellMarker database (http://xteam.xbio.top/CellMarker/). Subsequently, The proportion of different cell types in the dataset is evaluated by assessing the distribution of cell types across clusters.

### 2.3 Differentiation and enrichment analysis

To identify Differentially Expressed Genes (DEGs) within each cell type, we employed the “FindAllMarkers” function in the Seurat package. This analysis was based on the Wilcoxon rank-sum test with default parameters. We focused on clusters displaying logFC (fold change) values exceeding 0.25 and genes expressed in over 25% of the cells within the respective cluster. In order to gain a comprehensive understanding of the functional attributes associated with each identified cell type, we performed enrichment analysis on the DEGs utilizing the “clusterProfiler” R package (version 0.1.1).

### 2.4 Subpopulation analysis of oligodendrocytes cells

In order to investigate the heterogeneity within Oligodendrocytes, we performed a series of analyses. Initially, we isolated Oligodendrocyte cells and subsequently applied renormalization techniques to identify the top 2000 genes exhibiting high variability. Subsequently, we applied data normalization to ensure consistency across samples. To alleviate batch effects between samples, we utilized the harmony method during principal component analysis (PCA). This approach helped to remove any unwanted variation attributed to different experimental batches. We then selected the top 30 principal components (PCs) for downstream analysis, which involved downsampling and clustering of the cells.To visualize and explore the heterogeneity among the Oligodendrocyte cells, we employed the UMAP method. This technique facilitated the projection of the cells onto a two-dimensional map, enabling the identification of distinct subpopulations within the Oligodendrocyte population.

### 2.5 InferCNV identifies malignant cells

To discriminate malignant cells from non-tumor cells, we leveraged CNV analysis. Copy number variability within cell subpopulations was determined using the inferCNV algorithm. In this analysis, vascular endothelial cells served as the reference, allowing us to identify subpopulations exhibiting high copy number variability, which were designated as glioblastoma (GBM) cells.

### 2.6 Difference analysis and enrichment analysis of cell subpopulations

Next, we employed the “FindAllMarkers” function, employing the Wilcoxon rank-sum test, to detect genes that displayed differential expression within each subpopulation. Notably, our analysis was particularly concentrated on the Oligodendrocytes cell subpopulation. To gain deeper insights into the functional characteristics associated with these identified differentially expressed genes, we performed a Gene Ontology Biological Process (GO-BP) enrichment analysis using clusterProfiler.

### 2.7 Trajectory analysis

We employed a comprehensive approach utilizing three software packages to assess the developmental dynamics of differentiation within the Oligodendrocytes cell subpopulation.

Initially, cytoTRACE algorithm was utilized to evaluate the stemness of individual cell subpopulations. This assessment provided valuable insights into the cellular state and potential for differentiation.

Subsequently, Monocle R package (version 2.24.0) was employed for reconstructing cell differentiation trajectories. The DDRTree algorithm was utilized to construct these trajectories, while the FindVairableFeatures method and downscaling were employed to observe the developmental progression of subpopulation cells along the newly established trajectories (36).

To further analyze the cellular trajectories during glioblastoma multiforme (GBM) differentiation, we utilized the Slingshot package (version 2.6.0). This package facilitated the fitting of a minimum spanning tree (MST) to infer cell lineages using the getLineages function. Additionally, the getCurves function was employed to estimate the temporal changes in cellular expression levels within each lineage over time (37).

### 2.8 Cell communication

To elucidate the intricate communication between different cell subpopulations within GBM tumor tissues and their surrounding microenvironment, we employed the Cellchat R package (version 1.6.1) as a powerful tool (38). For this analysis, we utilized CellchatDB.human as a reference database for ligand-receptor interactions. By leveraging this computational framework, we deciphered cell-cell communication events at both the signaling pathway and receptor-ligand levels. This enabled us to gain insights into the coordinated interplay of signaling pathways across diverse cell types.

### 2.9 Modeling the prognosis of cancer-associated glioma cells

To specifically investigate the predictive value of GBM-associated cells in determining patient survival, we employed key subpopulation marker genes associated with GBM as predictive gene features. Using the “survival” R package, we employed both univariate Cox analysis and lasso regression analysis to identify the most significant prognostic genes. Subsequently, a prognostic model was developed through multivariate Cox analysis, incorporating the crucial genes identified from the previous analyses.

To derive the risk score for each sample, we computed it using the following formula: Riskscore = Expression of gene 1 multiplied by coefficient 1, plus Expression of gene 2 multiplied by coefficient 2, up to Expression of gene n multiplied by coefficient n.

Samples were classified into high-scoring and low-scoring groups based on the median value. Subsequently, survival analysis was performed to evaluate the prognostic outcomes of patients belonging to these distinct groups. For the purpose of assessing the performance of our model, we utilized the timeROC software package (version 0.4.0) to generate ROC curves at intervals of 1, 3, and 5 years.

Furthermore, in order to gain a more comprehensive understanding of the connection between gene expression patterns and patient outcomes, we investigated the correlation between the identified model genes, risk scores, and OS.

### 2.10 Assessment of tumor-infiltrating immune cells

To comprehensively evaluate the immune microenvironment of patients, we utilized a combination of CIBERSORT, ESTIMATE, and Xcell algorithms to calculate various immune-related scores. These scoring systems provided us with a comprehensive assessment of the patient’s immune status. Subsequently, under the CIBERSORT algorithm, we examined the high and low levels of 22 immune cells in different patient groups. Additionally, we conducted an investigation into the interrelation among the immune cells, risk scores, model genes, and OS. Furthermore, we examined the discrepancies in Stromal Score, Immune Score, ESTIMATE Score, and Tumor Purity among distinct patient cohorts. This thorough exploration allowed us to gain comprehensive insights into the intricate connections between these factors and patient outcomes.

### 2.11 Differential and enrichment analysis of bulk data

We employed the DESeq2 R package for conducting separate differential analyses on high and low-risk groups. To identify significant differences, we applied a threshold of |logFC|>2 with a p-value lower than 0.05.

In addition, we employed the clusterProfiler package to carry out GO, KEGG, and GSEA on the differentially expressed genes that were identified (39, 40).

### 2.12 Somatic mutation analysis

For somatic mutation analysis, we accessed the TCGA database to obtain the mutation data. We investigated the distribution of mutations in highly mutated genes as well as in modeled genes. To assess the tumor mutational burden (TMB) of each glioma sample, we employed the “maftools” software package. This computational tool allowed us to quantitatively measure the number of somatic mutations present in the genomic data of each tumor sample. Following this, we categorized the glioma samples into two distinct groups, distinguished as high TMB and low TMB groups, using the median TMB value. To evaluate the influence of TMB on survival outcomes, we applied the Kaplan-Meier method to compare the differences in survival between these two groups. This analysis allowed us to evaluate the potential prognostic significance of TMB in glioma patients. Furthermore, we examined the copy number variation (CNV) profile of the modeled genes. This enabled us to gain insights into potential genomic alterations associated with these genes and their possible implications in glioma development and progression.

### 2.13 Drug sensitivity analysis

To determine the IC50 (half-maximal inhibitory concentration) of chemotherapeutic drugs and assess their sensitivity in various groups, we utilized the pRRophetic R package (version 0.5) (41, 42).

### 2.14 Cell culture

The U-87 and LN229 cell lines, obtained from the Cell Resource Center of Shanghai Life Sciences Institute, were cultured in DMEM medium (Gibco BRL, USA). The cells were incubated at a temperature of 37°C in a humidified atmosphere enriched with 5% CO2. Furthermore, the culture medium was supplemented with 10% fetal bovine serum (FBS) acquired from Gibco BRL, a well-known supplier based in the United States. As the cells reached confluency, they were detached from the culture vessel using enzymatic or non-enzymatic cell dissociation methods. Subsequently, the dislodged cells were transferred into fresh culture flasks at an optimal cell density to facilitate subsequent proliferation and experimental investigations.

### 2.15 Cell transfection

Two distinct small interfering RNAs (siRNAs) designed to specifically target CRYAB were synthetized by Ribobio (Guangzhou, China). Transfections were performed utilizing Lipofectamine 3000 (Invitrogen, USA) following the manufacturer’s protocol. The siRNA sequences targeting CRYAB can be found in **Supplementary Table 1**.

### 2.16 RT-qPCR analysis

RNA extraction from cellular lines was conducted using TRIzol reagent (Thermo, 15596018) following established protocols (43, 44). Following RNA extraction, complementary DNAs (cDNAs) were generated by employing the PrimeScriptTM RT kit (Vazyme, R232-01). To quantify gene expression, the SYBR qPCR Master Mix (Vazyme, Q111-02) was utilized on the Roche LightCycler 480 (Roche, GER). Data analysis was carried out using the 2^−ΔΔCt^ method for gene expression analysis. The specific primer sequences, sourced from Tsingke Biotech (Beijing, China), can be found in **Supplementary Table 1**. GAPDH was employed as the internal reference gene for normalization purposes.

### 2.17 The experiment of cell-cunting-kit-8 assay

Cells were plated in 96-well plates at a density of 1 × 10^3^ cells per well, following standard protocols (45, 46). Following that, the plates were incubated in darkness at 37°C for 2 hours with CCK-8 labeling reagent (A311-01, Vazyme). The assessment of cell viability was carried out by measuring the absorbance at 450 nm using an enzyme-linked spectrophotometer (A33978, Thermo) at time intervals of 0, 24, 48, 72, and 96 hours.

### 2.18 The experiment of colony formation

A cohort comprising 1000 cells was transfected and cultured in 6-well plates for approximately 14 days. After a span of 2 weeks, the cellular clones were visually examined without magnification. Following that, the cells were washed and fixed using a 4% paraformaldehyde (PFA) solution for 15 minutes. Subsequently, the cells were subjected to crystal violet staining (Solarbio, China) for 20 minutes, and the samples were air-dried at room temperature. Finally, quantification of cells per well was conducted.

### 2.19 The experiment of wound healing

The transfected cells were cultured in 6-well plates and placed in a cell incubator until they reached a confluency level of 95%. A 200μl pipette tip was used to make a linear scratch on the cell monolayer. After washing off unattached cells and debris with PBS, the cells were transferred to a serum-free culture medium. Following that, images were taken at identical positions before and after 48 hours, and the width of the scratch was quantified using Image J software.

### 2.20 The experiment of transwell

Cell migration assays utilized Transwell chambers in the experimental setup. In each well of the upper compartment, 2×104 cells were seeded using a 200 μL serum-free medium. To evaluate cell migration, the upper region of the chamber was subjected to different conditions: in some cases, it was treated with Matrigel solution (BD Biosciences, USA), while in others it was left untreated. Following a 48-hour incubation period, the chambers were retrieved. The cells were subsequently fixed using 4% PFA and stained with 0.1% crystal violet (Solarbio, China). Subsequently, cell counting was carried out utilizing a light microscope. The migrated cells were photographed and quantified.

### 2.21 Apoptotic rate assessed through flow cytometric analysis

Apoptosis analysis was conducted utilizing an Annexin V-FITC/PI Apoptosis Detection Kit (Yeasen, Shanghai, China) according to the instructions provided by the manufacturer. Subsequently, flow cytometry (Cytoflex, Beckman, CA, USA) was employed to analyze the samples. The determination of apoptotic rate involved the consideration of both early apoptotic cells and terminal apoptotic cells.

### 2.22 Statistical analysis

For biological analysis in the field of medicine, R software version 4.1.3 was utilized, whereas GraphPad Prism version 8.0 was employed specifically for experimental data analysis. Mean values and standard deviations of the outcomes were extracted from three independent studies. Student’s t-tests were employed for pairwise comparisons between two groups, whereas comparisons involving more than two groups were evaluated using one-way ANOVAs followed by Tukey’s test. Significant differences were denoted as *P<0.05, **P<0.01, and ***P<0.001.

## 3 Results

### 3.1 snRNA sequencing reveals major cell types during GBM progression

To explore the cellular heterogeneity within the tumor microenvironment, we performed single-nucleus RNA sequencing (snRNA-seq) analysis on tumor specimens derived from 10 individuals diagnosed with GBM. After applying quality control and filtering criteria, we successfully profiled gene expression in 15,419 individual cells.

**Figure 1:**
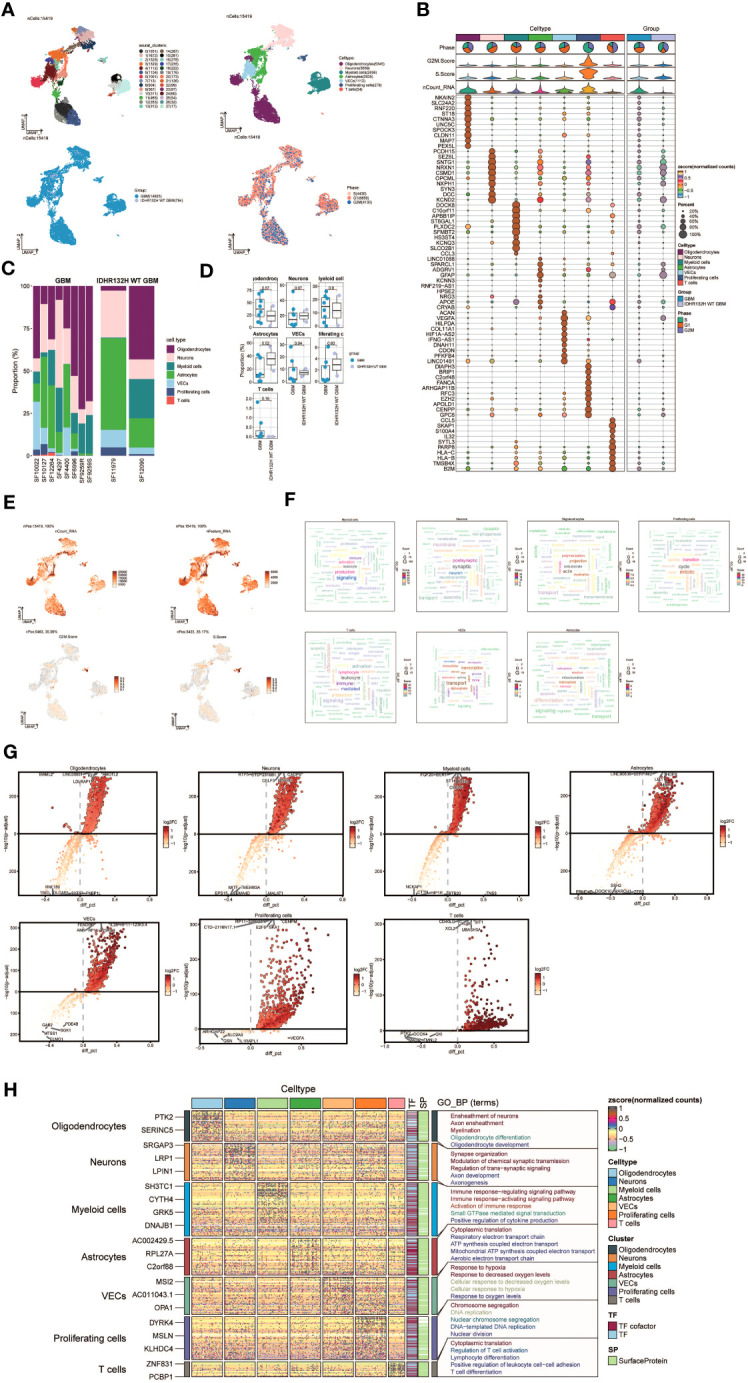
snRNA sequencing reveals major cell types during GBM progression. **(A)** UMAP plot showing the 28 clusters of cells in glioma patients and the number of cells in each cluster (top left); UMAP plot showing the 7 major cell types (top right); UMAP plot showing the distribution of the two groups of GBM and IDHR132H WT GBM for the 7 cell types (bottom left); and UMAP plot showing the distribution of different cell cycle phases (lower right). **(B)** Bubble plot showing differential expression of Top10maker genes in glioma cells across cell types. The color of the bubbles is based on the normalized data and the size indicates the percentage of genes expressed in the subpopulation. **(C)** Bar graph showing the percentage of the 7 cell types in the GBM group versus the IDHR132H WT GBM group. **(D)** Box line plot depicting the percentage of the 7 cell types in the GBM group versus the IDHR132H WT GBM group. **(E)** The UMAP plot showcases the distribution of the following relevant features: nCount_RNA, nFeature_RNA, S.score, and G2M.score. **(F)** Word cloud graph demonstrating gene pathway enrichment in the 7 cell types. **(G)** Volcano plot demonstrating differential gene expression in 7 cell types. **(H)** GO-BP enrichment analysis demonstrating biological processes associated with the 7 cell types.

Applying dimensionality reduction followed by clustering analysis, we identified 28 distinct cell clusters (**Figure 1A**, upper left), which were further classified into seven major cell types: Oligodendrocytes (5345 cells), Neurons (3559 cells), Myeloid cells (2436 cells), Astrocytes (2635 cells), Vascular Endothelial Cells (VECs) (1112 cells), Proliferating cells (278 cells), and T cells (54 cells) (**Figure 1A**, upper right). Among the 15,419 cells analyzed, 14,625 cells were derived from GBM lesions, while 794 cells originated from IDH R132H wild-type GBM lesions (**Figure 1A**, bottom left). Furthermore, we examined the distribution of these cell types within the cell cycle phases, revealing the following proportions: S phase (4430 cells), G1 phase (6859 cells), and G2M phase (4130 cells) (**Figure 1A**, bottom right).

To evaluate the expression patterns of marker genes across distinct cell types within the tumor, we generated bubble plots to showcase the top 10 marker genes for each identified cell population (**Figure 1B**). Moreover, we constructed a bar chart to depict the proportions of the seven cell types across eight patients with GBM lesions and two patients with IDH R132H wild-type GBM lesions, emphasizing the inter-patient heterogeneity in cell composition (**Figure 1C**). These findings underscore distinct cellular dynamics within GBM patients. Additionally, box plots were employed to demonstrate differential expression patterns across the seven cell types in different experimental groups (**Figure 1D**).

To offer a comprehensive dataset overview, we utilized UMAP plots to visualize the distribution of several key parameters for all cells, including nCount_RNA, nFeature_RNA, S score, and G2M score (**Figure 1E**). To uncover enriched Gene Ontology Biological Process (GO-BP) terms that are unique to individual cell types, we generated word clouds (**Figure 1F**). We visualized the differential gene expression analysis across cell types through volcano plots (**Figure 1G**). Moreover, we utilized a heatmap to illustrate the outcomes of GO-BP enrichment analysis for the differentially expressed genes across the seven cell types (**Figure 1H**).

### 3.2 Displaying the intracellular heterogeneity of oligodendrocytes

**Figure 2:**
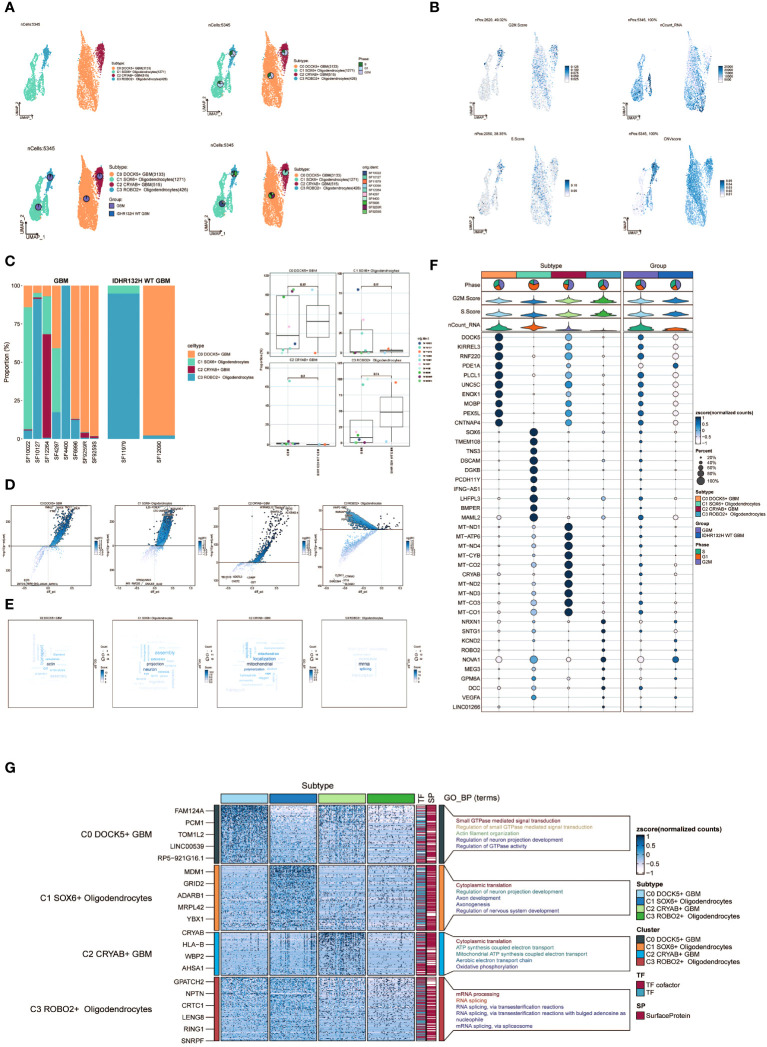
Visualization of oligodendrocytes cell subpopulations. **(A)** UMAP diagram demonstrating the 4 cell subpopulations of tumor cells in glioma patients and the number of cells in each subpopulation (top left); UMAP diagram demonstrating the percentage of different cell cycles in the 4 cell subpopulations (top right); UMAP diagram demonstrating the distribution of the GBM group versus the IDHR132H WT GBM group in the 4 cell subpopulations (bottom left); and UMAP diagram demonstrating the patient origin of the 4 cell subpopulations (lower right). **(B)** UMAP plot visualizing the relevant features of the 4 cell subpopulations: CNVscore, nCount_RNA,S.score,G2M.score. **(C)** Bar graph demonstrating the percentage of the 4 cell subpopulations in the GBM group versus the IDHR132H WT GBM group (left); box line graph depicting the percentage of the 4 cell subpopulations in the GBM group versus the IDHR132H WT GBM group (right). **(D)** Volcano plot demonstrating the expression of differential genes in the 4 cellular subpopulations. **(E)** Word cloud graph demonstrating gene pathway enrichment in the 4 cell subpopulations. **(F)** Bubble plot showing differential expression of Top10maker genes in 4 cell subpopulations. The color of the bubbles is based on the normalized data and the size indicates the percentage of genes expressed in the subpopulation. **(G)** GO-BP enrichment analysis demonstrating biological processes associated with the 4 cell subpopulations.

Following the implementation of dimensionality reduction clustering, we successfully discerned the presence of four distinct subgroups within oligodendrocytes. To distinguish between normal and cancer cells within GBM tissues based on genomic copy number variations (CNVs), we applied the inferCNV algorithm to explore single-cell data. Based on the inferCNV results, we categorized cells with high CNV levels as GBM cells (**Supplementary Figure 1**). This led to the identification of four cell subpopulations: C0 DOCK5+ GBM (3133 cells), C1 SOX6+ Oligodendrocytes (1271 cells), C2 CRYAB+ GBM (515 cells), and C3 ROBO2+ Oligodendrocytes (426 cells) (**Figure 2A**, upper left). UMAP plots were generated to visually represent the distribution and relative proportions of the four cell subpopulations based on cell cycle staging (**Figure 2A**, upper right), subgrouping (**Figure 2A**, lower left), and patient samples (**Figure 2A**, lower right).

Furthermore, we visualized several relevant features including CNV score, nCount_RNA, S score, and G2M score of the four cell subpopulations using UMAP plots (**Figure 2B**). Furthermore, the proportions of the four cell subgroups were assessed in a cohort of eight patients with GBM lesions and two patients with IDH R132H wild-type GBM lesions (**Figure 2C**, left). In our investigation, we observed an elevated prevalence of the C2 CRYAB+ GBM subgroup in the SF12264 patient. Nevertheless, statistical analysis utilizing box plots demonstrated no noteworthy variances in the proportions of these four subgroups among the different groups (**Figure 2C**, right). Volcano plots were employed to visually depict the distinct gene expression patterns among the four subpopulations (**Figure 2D**). Moreover, word cloud plots were generated to depict the Gene Ontology Biological Process (GO-BP) enriched pathway entries specific to each of the four subpopulations (**Figure 2E**). Bubble plots were employed to showcase the divergence in marker gene expression between Oligodendrocytes and GBM cells by highlighting the top 10 marker genes for each subpopulation (**Figure 2F**). In addition, heatmaps were generated to visually depict the outcomes of the Gene Ontology Biological Process (GO-BP) enrichment analysis pertaining to the differentially expressed genes observed within the four respective subpopulations (**Figure 2G**).

### 3.3 Visualization of pseudotime analysis of oligodendrocytes and GBM cell subpopulations by cytotrace and monocle

**Figure 3:**
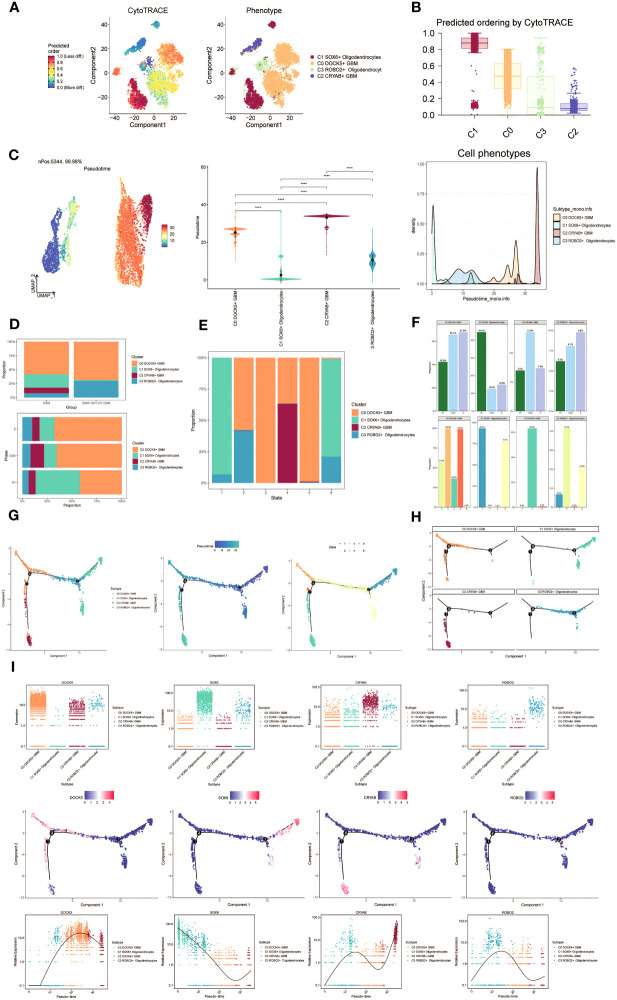
Visualization of proposed time series analysis of oligodendrocyte subpopulation and glioma cell subpopulation by cytotrace and monocle. **(A)** The differentiation of oligodendrocyte subpopulation and glioma cell subpopulation is analyzed using cytotrace and displayed in a 2D graph. The color can represent the level of differentiation. The figure on the right represents the cytotrace results displayed according to different oligodendrocyte subpopulations and glioma cell subpopulations. The colors represent different cell subpopulations. **(B)** Box line plot showing the visualization results of cytotrace analysis of oligodendrocyte subpopulations and glioma cell subpopulations. **(C)** UMAP plots, violin plots and ridge plots showing the pseudotime distribution of oligodendrocyte subpopulations and glioma cell subpopulations. *p ≤ 0.05, **p < 0.0 1, ***p < 0.001, ****p < 0.0001. indicates a significant difference, ns indicates a non-significant difference. **(D)** The occupancy of relevant features in different pseudotime stages of 4 cell subpopulations was visualized: the occupancy of 4 cell subpopulations in the GBM group vs the IDHR132H WT GBM group (top); the occupancy of 4 cell subpopulations in different cell cycles (bottom). **(E)** Bar charts illustrating the proportions of different pseudotime stages (state1-state6) within the four cell subgroups. **(F)** Bar graph demonstrating the expression of the 4 cell subpopulations in different phases (top) vs. different states (bottom), respectively. **(G)** Demonstrating the derivation process of oligodendrocyte subpopulation and glioma cell subpopulation. Left: UAMP plot of the proposed temporal trajectory showing the 4 cell subpopulations; Middle: UMAP plot showing the pseudotime trajectory of oligodendrocyte subpopulation and glioma cell subpopulation, starting from the upper right and dividing into two branches, one of which differentiates downward and the other to the left followed by two more branches, one of which differentiates upward to the left, and the other downward; Right: UMAP plot showing the distribution of 6 states on the proposed temporal trajectory plot. **(H)** Split-plane plots of the proposed temporal trajectories of oligodendrocyte subpopulations and glioma cell subpopulations showing the distribution of different cell subpopulations on the proposed temporal trajectories, respectively. **(I)** Scatter plot showing the changes of 4 cell subpopulations of oligodendrocyte subpopulation and glioma cell subpopulation with the proposed chronological sequence; proposed chronological sequence UMAP plot showing the changes of the cell subpopulations corresponding to the 4 named genes with the proposed chronological sequence; and the expression of the 4 named genes of cell subpopulations (DOCK5, SOX6, CRYAB, ROBO2) on the pseudotime trajectory.

To investigate the differentiation and developmental relationship among the four cell subpopulations, we performed an analysis of Oligodendrocytes and GBM cell subpopulations using cytotrace (**Figure 3A**). The results were visualized, demonstrating that the four cell subpopulations differentiated along the C1-C0-C3-C2 trajectory (**Figure 3B**).

To depict the differentiation trajectories of Oligodendrocytes and GBM cell subgroups, we utilized UMAP plots, violin plots, and ridge plots to visualize the four cell subgroups at the pseudotime level (**Figure 3C**). We observed a continuum of Oligodendrocytes and GBM cell subpopulations at the pseudotime level. The percentage of these four cell subpopulations was compared between the GBM group and the IDHR 132H WT GBM group using bar graphs (**Figure 3D**, top). It was noted that the percentage of C0 DOCK5+ GBM was higher in both groups, while the C2 CRYAB+ GBM subpopulation was exclusively found in the GBM group. This suggests a potential relationship between the GBM group and the IDHR 132H WT GBM group, which merits further investigation. Bar graphs were also employed to display the cell occupancy of the four cell subpopulations across different cell cycle stages (**Figure 3D**, bottom).

In the bar graph presented in **Figure 3E**, the distribution of cell percentage in the four cell subpopulations across different trajectory differentiation states was illustrated. Notably, the C1 SOX6+ Oligodendrocytes subpopulation showed a higher percentage in state1 and state6, while the C0 DOCK5+ GBM subpopulation was almost 100% present in state3 and state5. The C2 CRYAB+ GBM subpopulation exhibited a higher percentage in state4 and was almost absent in other states, which is of particular interest. We then provided detailed information on the percentage of cells in each cell subpopulation based on cell cycle phase (**Figure 3F**, top) and trajectory differentiation state (**Figure 3F**, bottom).

To further explore the origin of GBM cytogenesis, we conducted trajectory analysis using monocle analysis on the four cell subpopulations (**Figure 3G**). The analysis revealed a continuous distribution of the four cell subpopulations along a pseudotime trajectory with four branching points. The trajectory initiated from the upper-right region and reached the second differentiation point as state1, which further split into two branches. One branch extended downwards corresponding to state6, while the other branch continued differentiation towards the left, corresponding to state2. At the third differentiation point, another split occurred, with one branch differentiating towards the upper-left (state3) and the other branch differentiating downwards (state4). A short branch emerged towards the upper-left at the first differentiation point, corresponding to state5. According to the proposed chronology, the C1 SOX6+ Oligodendrocytes subpopulation corresponds to the early stages of tumor development and continues to differentiate into other subpopulations. It is likely that the C0 DOCK5+ GBM or C2 CRYAB+ GBM subpopulations represent further differentiation stages. The facets analysis of each subpopulation provided supporting evidence for this conclusion (**Figure 3H**).

Selected genes specific to the four cell subpopulations were examined, and their changes across the pseudotime series were visualized using scatter plots, pseudotime series UMAP plots, and pseudo-scatter plots (**Figure 3I**). The C1 cell subpopulation, represented by the SOX6 gene, predominantly appeared at the beginning of the proposed time series, while the C2 cell subpopulation, represented by the CRYAB gene, was primarily observed at the end of the pseudotime series.

These findings shed light on the differentiation and developmental patterns within the four cell subpopulations, providing insights into the origin and progression of GBM cells.

### 3.4 Slingshot analysis of oligodendrocytes and GBM cell subpopulations of pseudotime trajectories

**Figure 4:**
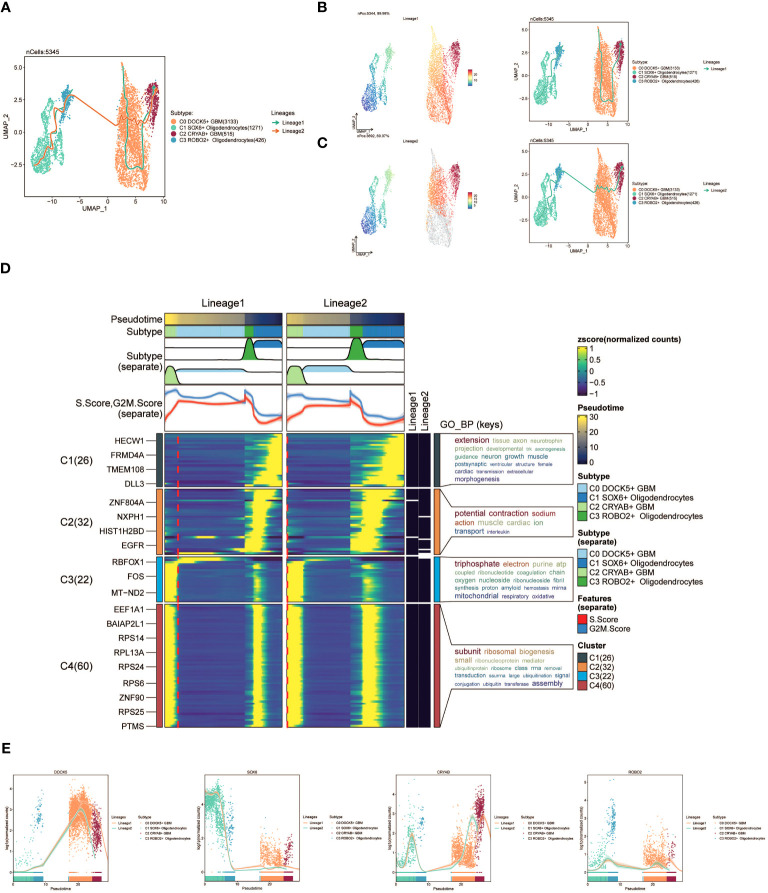
slingshot analysis of the pseudotime trajectories of oligodendrocyte subpopulations and glioma cell subpopulations. **(A)** UMAP plot showing the distribution of two differentiation trajectories of oligodendrocyte subpopulation and glioma cell subpopulation fitted by the pseudotime order in all cells. **(B)** UMAP plot demonstrating the change of Lineage1 with the fitted temporal order (left); UMAP plot demonstrating the differentiation trajectory of Lineage1 on the fitted temporal order (right). **(C)** UMAP plot demonstrating the change of Lineage2 with the fitted temporal order(left); UMAP plot demonstrating the differentiation trajectory of Lineage2 on the fitted temporal order (right). **(D)** GO-BP enrichment analysis demonstrating the biological processes corresponding to the two proposed chronological trajectories of oligodendrocyte subpopulation and glioma cell subpopulation. **(E)** Scatterplot demonstrating the trajectories of the named genes of the four cell subpopulations of oligodendrocyte subpopulation and glioma cell subpopulation obtained after slingshot visualization.

Furthermore, we employed slingshot to further analyze the cellular trajectories during GBM differentiation. Initially, we illustrated the trajectories of the four cell subpopulations (C0 to C3), revealing a continuous distribution along the temporal axis with differentiation into two distinct lineages (**Figure 4A**). We estimated the pseudotime sequences at the cellular level along these two lineages (**Figure 4B, C**). Lineage1 originated from C0 and progressed through C2, while lineage2 commenced from C1 and progressed through C3 before shifting to C0 and C2.

To gain insights into the biological processes associated with the two pseudotime trajectories, we performed GO-BP enrichment analysis. We discovered that C1, present in both lineage1 and lineage2, exhibited associations with various biological processes, including extension, tissue development, and axon growth. C2 was linked to biological processes like potential, fraction, and sodium. C3 exhibited associations with biological processes such as triphosphate and electron, while C4 was related to subunit and ribosomal processes (**Figure 4D**).

Lastly, scatter plots were utilized to visually represent the distribution of distinct cell subpopulations along the lineage1 and lineage2 trajectories, portraying their respective differentiation curves throughout the pseudotime series (**Figure 4E**).

### 3.5 Cellchat analysis between cells and visualization of PSAP signaling pathways

**Figure 5:**
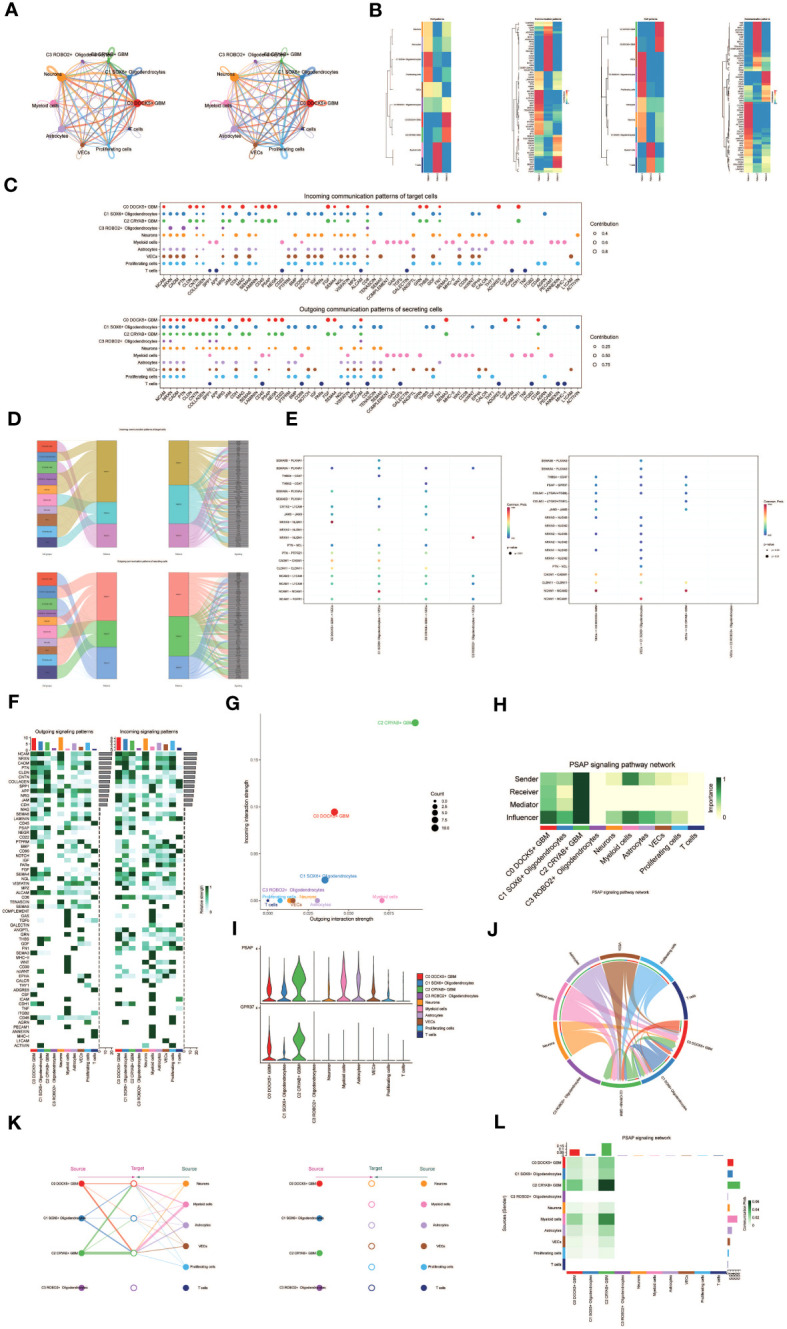
Cellchat results presentation. **(A)** Circle plot showing the number (left) and strength (right) of interactions between all cells. **(B)** Heatmap showing pattern recognition of outcoming cells in all cells (left), and pattern recognition of incoming cells (right). **(C)** Outcoming contribution bubble plots and incoming contribution bubble plots showing the expression of cellular communication patterns between each cell subpopulation and other cells in the oligodendrocyte subpopulation and glioma cell subpopulation. **(D)** Mulberry diagram showing cellular communication patterns between all cells. Top: incoming Mulberry diagram, bottom: outcoming Mulberry diagram. **(E)** Scatterplot of cellular communication patterns for screening interactions between glioma cell subpopulations, oligodendrocyte subpopulations and vascular endothelial cells. The color of the dots indicates varying degrees of functional strength and the size of the dots indicates the number of cells. p-value < 0.01, statistically different. **(F)** Heatmap showing afferent and efferent signal intensities of all cell interactions **(G)** Scatter plot of cellular communication patterns of PSAP signaling pathway. **(H)** Centrality score of PSAP signaling pathway network demonstrated by heatmap. **(I)** Violin plot of cellular interactions in the PSAP signaling pathway. **(J)** Circle plot of cellular interactions in the PSAP signaling pathway with oligodendrocyte subpopulation and glioma cell subpopulation as RECEIVER. **(K)** Hierarchical diagram of oligodendrocyte subpopulations and glioma cell subpopulations interacting with other cells in the PSAP signaling pathway. **(L)** Interaction of cells in the PSAP signaling pathway shown by heatmap.

To systematically unravel the intricate cellular responses, we aimed to investigate cell-to-cell relationships and ligand-receptor communication networks, ultimately enhancing our understanding of intercellular interactions. By employing Cellchat analysis, we initially constructed intercellular communication networks among various cell types, including Oligodendrocytes, subpopulations of GBM cells, Myeloid cells, Astrocytes, T cells, and others. We quantified the cellular interplay, measuring the frequency of interactions between different cell types (illustrated by the thickness of the connecting lines) and the intensity of these interactions (indicated by the weight of the lines) (**Figure 5A**).

Besides examining individual signaling pathways, gaining insight into the coordination of functions among multiple cell populations and signaling pathways was essential. To tackle this issue, Cellchat employed a pattern recognition technique utilizing non-negative matrix decomposition. This approach aimed to identify overall communication patterns and critical signaling molecules within distinct cellular clusters. The results of this analysis yielded communication patterns that connected cell populations with signaling pathways in the context of efferent signaling (cells acting as senders) or afferent signaling (cells acting as receivers). We utilized gene expression pattern analysis provided by Cellchat to unveil interactions between cells and signaling pathways.

Through this analysis, we identified three efferent signaling patterns and three afferent signaling patterns. Each efferent communication pattern was associated with a specific cell type predominantly responsible for that pattern: pattern 1 (Oligodendrocytes, VECs, Proliferating cells), pattern 2 (Myeloid cells), and pattern 3 (a subpopulation of GBM cells) (**Figure 5B**). For instance, we observed that GBM efferent signaling was primarily characterized by mode 3, encompassing multiple pathways such as CNTN and MAG, among others. T cell and myeloid cell signaling were predominantly represented by mode 2, which included pathways like CD45, CD99, and GAS. On the other hand, the afferent signaling pattern indicated that GBM afferent signaling was predominantly associated with mode 3, incorporating pathways like CD45, PSAP, and others (**Figure 5D**).

To identify key afferent and efferent signals associated with the four cell subpopulations, we quantitatively assessed ligand-receptor networks using Cellchat’s pattern recognition methods. In GBM, each cell type could act as a signal sender, releasing various cell factors or ligands, while also functioning as a signal receiver, with receptors targeted by ligands from the same or different cell types. The ligand-receptor-mediated communication across different cell types was anticipated to contribute to GBM development (**Figure 5C**). Specifically, we focused on the communication between Oligodendrocyte subpopulations and GBM cell subpopulations with vascular endothelial cells, demonstrating their ligand-receptor relationships (**Figure 5E**).

To depict the incoming and outgoing signal strengths of all cell interactions, we presented a heatmap (**Figure 5F**). To investigate the PSAP signaling pathway’s mechanism of action, we visualized and analyzed the pathway. Scatter plots were employed to demonstrate the cellular communication pattern of the PSAP signaling pathway, revealing the prominence of the GBM cell subpopulation C2 CRYAB+ GBM within this pathway (**Figure 5G**). Additionally, we identified cell types as mediators and influencers of intercellular communication in the PSAP signaling pathway based on a centrality measure algorithm. Notably, the GBM cell subpopulation C2 CRYAB+ GBM exhibited the highest importance in the PSAP signaling pathway (**Figure 5H**). A violin plot illustrated cell-cell interactions and highlighted the high expression of the PSAP signaling pathway in the GBM subpopulation C2 CRYAB+ GBM, suggesting its significance within the context of GBM (**Figure 5I**). Chordal plots were employed to demonstrate receptor-ligand profiles of GBM cell subpopulations and Oligodendrocyte cell subpopulations with other intercellular receptors (**Figure 5J**).

By considering all ten identified cell types in GBM tissues as the source cells of the PSAP signaling pathway and selecting specific cell types as potential target cells, we utilized layered diagrams to visualize potential targets of PSAP released from different cell types. Our results indicated that PSAP released from eight cell types could potentially target the C0 DOCK5+ GBM subpopulation, the C1 SOX6+ Oligodendrocytes subpopulation, and the C2 CRYAB+ GBM subpopulation, while PSAP released from the other six cell types did not target any specific cells (**Figure 5K**). Our findings suggested that all cell types except C3 ROBO2+ Oligodendrocytes and T cells might act as signal emitters in the PSAP signaling pathway. Detailed intercellular interactions within the PSAP signaling pathway were presented (**Figure 5L**).

### 3.6 Establishment and validation of the prognostic model

**Figure 6:**
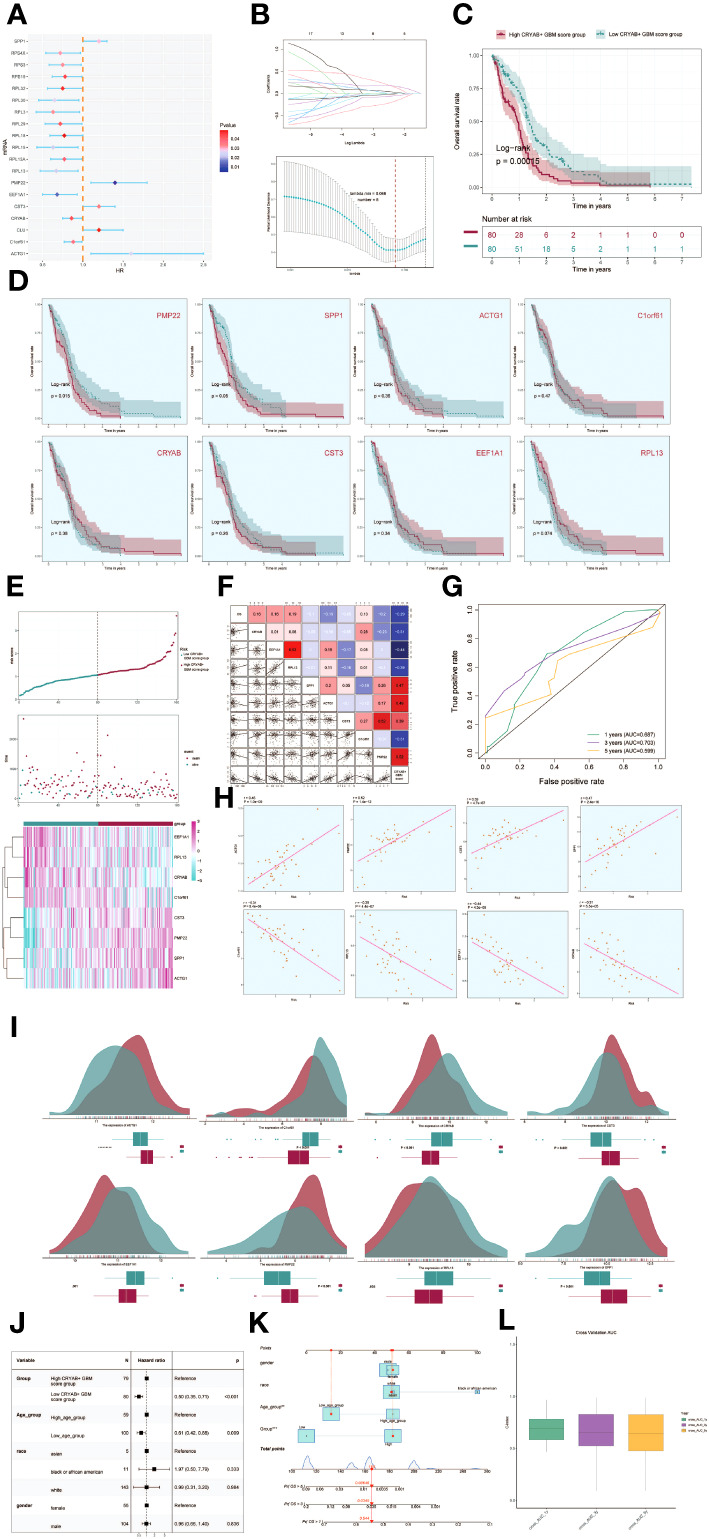
Development of a prognostic model associated with CRYAB+ GBM scores. **(A)** Forest plot showing univariate cox analysis of genes constituting CRYAB+ GBM score. Null line HR=1, HR<1 protective factor, HR>1 risk factor. **(B)** 8 genes that constitute CRYAB+ GBM score screened by lasso regression (top); Lambda plot of genes that constitute CRYAB+ GBM score (right). **(C)** Survival analysis status of the screened 8 genes constituting CRYAB+ GBM score in both high and low CRYAB+ GBM score groups. **(D)** Survival analysis plot of the 8 genes constituting the high and low CRYAB+ GBM score groups. **(E)** Curve plots showing hazard scores in the high and low CRYAB+ GBM score groups (top); scatter plot illustrates survival status variations between high and low CRYAB+ GBM score groups(middle); heatmaps showing gene expression of genes constituting the high and low CRYAB+ GBM score groups, with color scales based on normalized data (bottom). Green indicates the low CRYAB+ GBM score group and red indicates the high CRYAB+ GBM score group. **(F)** Correlation analysis between CRYAB+ GBM scores, overall survival (OS), and genes used in model establishment. Red indicates positive correlation, blue indicates negative correlation, and color shades indicate high or low correlation. **(G)** AUC scores for 1, 3, and 5 years are shown by ROC plot. AUC(1-year): 0.687, AUC(3-year):0.703, AUC(5-year):0.599. **(H)** Scatter plot showing the correlation analysis of the genes constituting CRYAB+ GBM score with CRYAB+ GBM score. **(I)** Peak and box plot showing the difference in expression of the eight genes constituting CRYAB+ GBM score in the high and low CRYAB+ GBM score groups. **(J)** Forest plot showing multivariable Cox regression analysis of CRYAB+ GBM score in conjunction with other clinical factors. Null line HR=1, HR<1 protective factor, HR>1 risk factor. **(K)** Nomogram plots predicting OS (overall survival) at 1, 3, and 5 years based on age, high and low CRYAB+ GBM score subgroups, and stage. **(L)** Box line plot for internal cross validation of AUC scores at 1, 3, and 5 years.

To evaluate the clinical significance of the identified cell types in this study, we conducted a univariate COX analysis on the top 100 marker genes for the C2 CRYAB+ GBM subgroup. This analysis revealed that 19 genes were associated with patient prognosis. Among them, SPP1, PMP22, CST3, CLU, and ACTG1 were identified as risk factors, while the remaining genes were found to be protective factors (P<0.05) (**Figure 6A**). To address the issue of multicollinearity among these genes, we conducted lasso regression to further refine the selection, resulting in a set of 8 genes that constituted the CRYAB+ GBM score. The Lambda plot validated this selection (**Figure 6B**).

Subsequently, subjects were stratified into two cohorts based on the gene expressions of the 8 chosen genes, namely the high CRYAB+ GBM score cohort and the low CRYAB+ GBM score cohort. Survival analysis was then conducted for both cohorts (**Figure 6C**). Results indicated superior survival outcomes in the low CRYAB+ GBM score group, and conversely, poorer survival outcomes in the high CRYAB+ GBM score group.

Survival analysis focusing on the eight genes comprising the CRYAB+ GBM score model revealed statistically significant results specifically for PMP22 and SPP1 (**Figure 6D**). Higher gene expression consistently associated with worse survival outcomes, while lower expression consistently correlated with improved prognosis, confirming their established role as risk factors.

The CRYAB+ GBM score was determined for each patient in the TCGA-GBM dataset, taking into account the expression levels of the eight genes and their corresponding regression coefficients). After plotting the distribution of CRYAB+ GBM scores in the TCGA-GBM dataset, the patients were classified into high and low score groups based on the median value. Furthermore, the survival time distribution demonstrated a negative prognostic impact associated with higher CRYAB+ GBM scores. The expression levels of the eight genes comprising the model were also visually depicted (**Figure 6E**).

Correlation analysis among survival days, CRYAB+ GBM score, and the genes in the model revealed a negative correlation between overall survival (OS) and CRYAB+ GBM score, a significant negative correlation between EEF1A1 and CRYAB+ GBM score, and positive correlations among most of the other modeled genes. The scatter plot further visualized the relationships among the eight modeling genes, CRYAB+ GBM score, and OS (**Figure 6F**).

In order to assess the predictive accuracy of the CRYAB+ GBM score for survival outcomes at 1, 3, and 5 years, ROC curves were generated, yielding corresponding area under the curve (AUC) values of 0.687 (1-year survival), 0.703 (3-year survival), and 0.599 (5-year survival) (**Figure 6G**). Scatter plots were employed to illustrate the associations between the genes included in the model and the CRYAB+ GBM score (**Figure 6H**). The variation in expression levels of the eight modeled genes between the high and low CRYAB+ GBM score groups was exhibited (**Figure 6I**).

To determine whether the CRYAB+ GBM score was an independent risk factor, we constructed a gene-cytotype clinical prediction model and performed multi-factorial Cox regression analysis, considering age, ethnicity, T-stage, N-stage, M-stage, and the high/low CRYAB+ GBM score groups as factors. The findings of our study indicated that the CRYAB+ GBM score exhibited statistical significance (p<0.05) as an independent prognostic risk factor for patients with GBM (**Figure 6J**). Age, ethnicity, and T-stage were subsequently used to construct a nomogram diagram that integrated clinical and pathological risk factors, as well as risk cell type characteristics. This nomogram diagram effectively predicted the probability of patients’ survival and displayed the 1-year, 3-year, and 5-year survival rates (**Figure 6K**).

To further validate the accuracy of the nomogram diagram, we internally cross-validated the results using a box-and-line plot (**Figure 6L**).

### 3.7 Immune infiltration patterns and differences between patients with high CRYAB+ GBM scores and those with low CRYAB+ GBM scores

**Figure 7:**
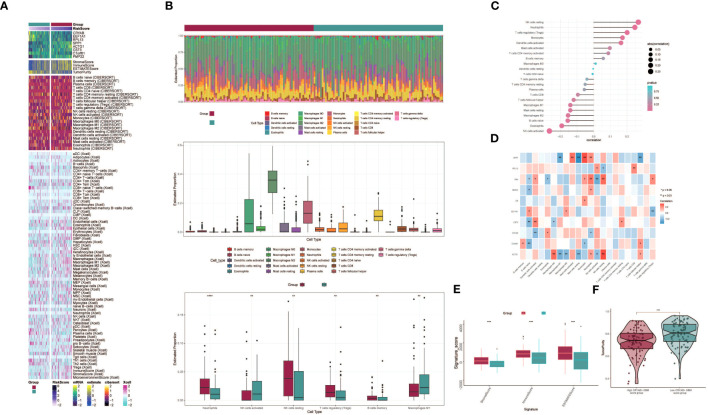
Differential analysis of immune infiltration in high and low CRYAB+ GBM score groups. **(A)** Heatmap shows the expression of various immune scores in high and low CRYAB+ GBM score groups. **(B)** Stacked bar graph of immune infiltration (top); box-and-line graph showing the expression of 22 immune cells in gliomas(middle); infiltration of 6 immune cells with significant differences in high and low CRYAB+ GBM score groups is shown by box-and-line graph (bottom). **(C)** The lollipop chart shows the correlation between immune cells and CRYAB+ GBM score. **(D)** Correlation of immune cells with genes constituting CRYAB+ GBM score is shown by bar graph, heat map. *p ≤ 0.05, **p < 0.0 1; ***p < 0.001 indicates a significant difference, ns indicates a non-significant difference. **(E)** Box line plots showing differences between high and low CRYAB+ GBM score groups in stromal score, immune score, and stromal and immune signature gene set scores. *p ≤ 0.05, **p < 0.0 1; ***p < 0.001 indicates a significant difference and ns indicates a non-significant difference. **(F)** Differences in tumor purity in high and low CRYAB+ GBM score groups are shown by violin plots. *p ≤ 0.05, **p < 0.0 1, ***p < 0.001, ****p < 0.0001. indicates significant difference, ns indicates insignificant difference.

To explore immune infiltration in GBM and its association with the two groups, we employed heatmaps as a visual representation of the distinct expression patterns of immune infiltration between the high CRYAB+ GBM score group and the low CRYAB+ GBM score group (**Figure 7A**). We subsequently utilized the CIBERSORT algorithm to estimate the proportions of 22 immune cell types in GBM patients sourced from the TCGA database. Our investigation primarily concentrated on discerning the immune infiltration status within the high CRYAB+ GBM score group and the low CRYAB+ GBM score group, resulting in the identification of the predicted composition of various immune cell subsets (**Figure 7B**, top).

Further analysis of the immune infiltration in the two groups highlighted the differences in the predicted abundances of six immune cell types. Within the high CRYAB+ GBM score group, increased levels of resting NK cells and regulatory T cells (Tregs) were observed. Conversely, the low CRYAB+ GBM score group displayed elevated proportions of M1 macrophages and activated NK cells (**Figure 7B**, bottom). Bar graphs were utilized to present the correlation between immune infiltrating cells and GBM subpopulation labeling scores. The results demonstrated a positive correlation between the CRYAB+ GBM score and resting NK cells, neutrophils, regulatory T cells (Tregs), among others. Conversely, a negative correlation was observed between the CRYAB+ GBM score and activated NK cells, eosinophils, naive B cells, among other cell types (**Figure 7C**).

To explore the relationships between the eight modeled genes and immune cells, we employed multiple methods of immune cell content assessment and presented the results in a heatmap. The heatmap represented positive correlations in red shades and negative correlations in blue shades (**Figure 7D**).

Moreover, we performed an assessment of the Stromal Score, Immune Score, and ESTIMATE Score in the high CRYAB+ GBM score group versus the low CRYAB+ GBM score group. The findings revealed elevated levels of the Stromal Score, Immune Score, and ESTIMATE Score in the high CRYAB+ GBM score group as opposed to the low CRYAB+ GBM score group (**Figure 7E**). Visualization of Tumor Purity was conducted for both cohorts, indicating a decreased level of Tumor Purity in the high CRYAB+ GBM score group when compared to the low CRYAB+ GBM score group (**Figure 7F**).

### 3.8 Analysis of differences and enrichment analysis

**Figure 8:**
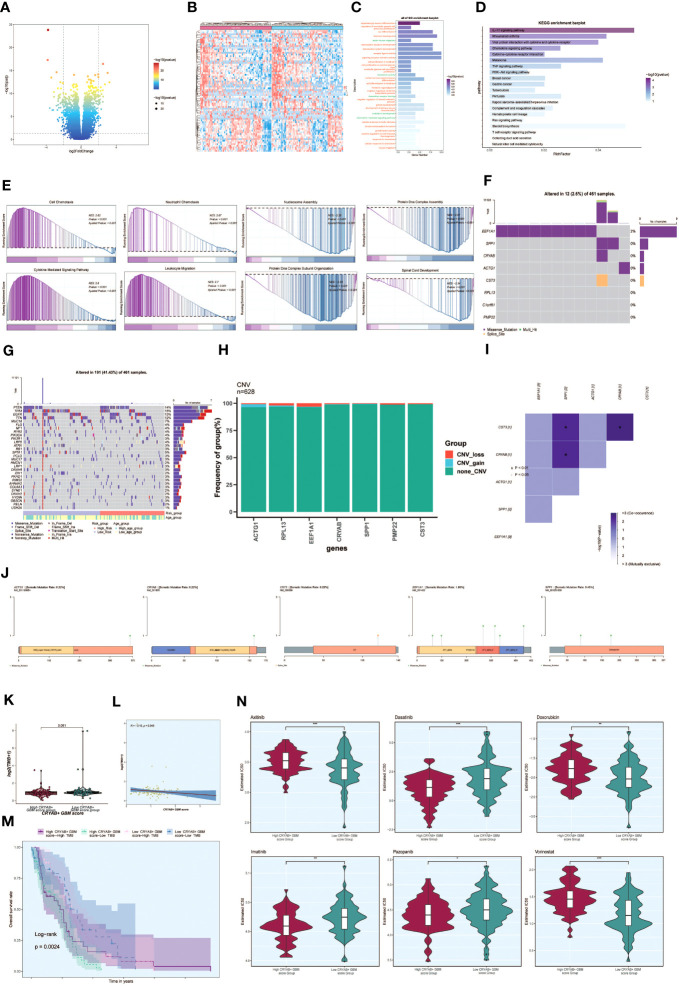
Enrichment analysis, mutation analysis and drug sensitivity analysis between different groups. **(A, B)** Volcano and heatmap showing the expression of differential genes in the high and low CRYAB+ GBM score groups. **(C)** Bar graph showing the results of all GO enrichment analyses (GOBP, GOCC, GOMF). **(D)** Results of enrichment on different pathways are shown by KEGG enrichment analysis of differential genes. **(E)** Showing the enrichment score values of genes on different pathways by GSEA scoring of GO-BP enrichment entries of differential genes. **(F)** Gene mutation waterfall plot showing mutations of the genes constituting the CRYAB+ GBM score in the samples. The top bar indicates the mutation load for each sample, and the right bar indicates the total percentage of mutations for that gene in those samples. **(G)** Mutation waterfall plot showing differences in the top 30 most frequently mutated genes in somatic cells between the two groups. The top bar indicates the mutation load for each sample, and the right bar indicates the total percentage of mutations in that gene in those samples. **(H)** CNV status of model genes **(I)** Heatmap showing the correlation between the mutation profiles of the genes that make up the CRYAB+ GBM score. **(J)** Visualization of the mutation profiles of different genes using lollipop plots. **(K)** Difference in mutation load in high and low CRYAB+ GBM score groups using violin plots. **(L)** Scatter plot showing the correlation analysis between mutation load and CRYAB+ GBM score. **(M)** Scoring according to tumor mutation load, divided into four groups: high-risk high mutation load, high-risk low mutation load, low-risk high mutation load, and low-risk low mutation load, and the curves show the survival analysis results of the four groups. **(N)** Differences in different drug sensitivities in high and low CRYAB+ GBM score groups are shown by violin plots. *, p ≤ 0.05; **p < 0.0 1; ***p < 0.001 indicates a significant difference, and ns indicates a non-significant difference.

To investigate the differences between the high CRYAB+ GBM score group and the low CRYAB+ GBM score group, we utilized volcano plots and heatmaps to visualize the expression of differentially expressed genes (**Figures 8A, B**). To gain a deeper understanding of the potential involvement of the C2 subgroup characterized by CRYAB+ expression in the initiation and progression of GBM, we performed functional enrichment analysis on the set of genes exhibiting differential expression between the two groups. The results of GO enrichment analysis were presented as bar graphs, showcasing associations with pathways such as dopaminergic neuron differentiation, regulation of cerebellar granule cell precursor proliferation, sex differentiation, and forebrain development (**Figure 8C**).

In addition, we conducted KEGG enrichment analysis on the set of differentially expressed genes and represented the outcomes through bar graphs, which unveiled significant enrichments in various pathways. These pathways encompassed the IL-17 signaling pathway, Rheumatoid arthritis, and Viral protein interaction with cytokine and cytokine receptor (**Figure 8D**). The enrichment scores for genes on different pathways were demonstrated through GSEA scoring of GO-BP-enriched entries of the differentially expressed genes (**Figure 8E**).

### 39 Mutation analysis

To explore the association between genetic mutations and immune components within the tumor microenvironment (TME), we performed supplementary investigations and visually represented the cellular mutation data obtained from both study cohorts. A model was used to display the mutations in the eight genes (**Figure 8F**). We compared the top 30 genes displaying the highest mutation frequencies in the interstitial cells of the two groups. The upper bars represent the mutation load for each sample, while the right bars indicate the total proportion of mutations in each gene within those samples (**Figure 8G**).

To assess chromosomal copy number variation (CNV) gain and loss, bar graphs were employed. However, the results revealed no significant chromosomal CNV gain or loss in the modeled genes (**Figure 8H**). Additionally, a heatmap displayed the correlation of mutation profiles among the genes comprising the CRYAB+ GBM score (**Figure 8I**). Furthermore, a lollipop plot was utilized to visualize the mutation profiles of different genes (**Figure 8J**).

Violin plots were utilized to examine the variation in mutation load between the high CRYAB+ GBM score group and the low CRYAB+ GBM score group. Nevertheless, no statistically significant differences were observed (**Figure 8K**). Scatter plots were employed to illustrate the statistical significance (p < 0.05) in the correlation between mutation load and the CRYAB+ GBM score (**Figure 8L**).

Furthermore, tumor samples were assessed and classified into four distinct groups based on their mutational load: High TMB with High CRYAB+ GBM score, Low TMB with High CRYAB+ GBM score, High TMB with Low CRYAB+ GBM score, and Low TMB with Low CRYAB+ GBM score. Survival analysis curves depicted the outcomes for these groups, with the Low CRYAB+ GBM score-Low TMB group demonstrating the best survival, while the High CRYAB+ GBM score-Low TMB group exhibited the worst survival (**Figure 8M**).

### 310 Drug sensitivity analysis

Violin plots were utilized to illustrate the variation in drug sensitivity between the high CRYAB+ GBM score group and the low CRYAB+ GBM score group (**Figure 8N**). Notably, we observed differential responses to specific drugs. For instance, Dasatinib, an FDA-approved CNS permeant for GBM, exhibited a higher IC50 value in the low CRYAB+ GBM score group compared to the high CRYAB+ GBM score group. This finding suggests that the high CRYAB+ GBM score group may display potentially greater sensitivity to the drug.

### 3.11 Knocking down CRYAB expression effectively suppresses the proliferation, migration, and metastatic capabilities of glioma cells

**Figure 9:**
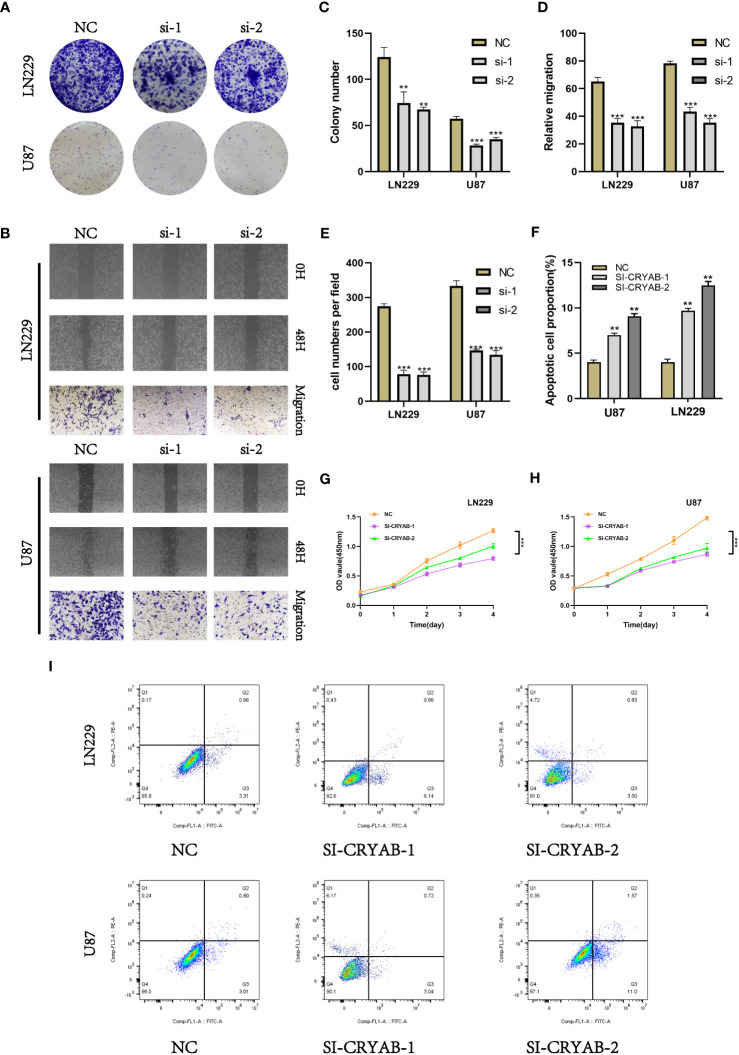
Silencing CRYAB Inhibits Proliferation, Migration and Metastasis while Promoting Apoptosis in Glioma Cells. **(A)** Colony formation assay was performed on U87 and LN229 glioma cells in the NC and si-CRYAB groups. Smaller colonies were observed in the si-CRYAB group, indicating that CRYAB silencing inhibits glioma cell proliferation. **(B)** Transwell assay demonstrated a decrease in the migration ability of U87 and LN229 cells in the si-CRYAB group compared to the NC group. And scratch assay revealed a decrease in the migration ability of U87 and LN229 cells in the si-CRYAB group compared to the NC group. **(C)** Quantification of colony formation assay results showing a decrease in colony size in the si-CRYAB group compared to the NC group. **(D)** Quantification of scratch assay results showing a decrease in wound closure percentage in the si-CRYAB group compared to the NC group. **(E)** Quantification of transwell assay results showing a decrease in the number of invading cells in the si-CRYAB group compared to the NC group. **(F)** Quantification of apoptosis assay results showing an increase in the percentage of apoptotic cells in the si-CRYAB group compared to the NC group. **(G)** CCK-8 assay further confirmed the inhibitory effect of CRYAB silencing on LN229 cells proliferation. **(H)** CCK-8 assay further confirmed the inhibitory effect of CRYAB silencing on U87 cells proliferation. **(I)** Apoptosis assay revealed an increase in apoptosis in both U87 and LN229 cell lines upon CRYAB silencing. **p < 0.01, ***p < 0.001.

To investigate the impact of CRYAB in glioma, we performed CRYAB gene transfection knockdown and the transfection efficiency was verified by RT-qPCR (**Supplementary Figure 2**). Then we conducted colony formation assays on U87 and LN229 glioma cells in the negative control (NC) and si-CRYAB groups (**Figure 9A**). The results indicated that the suppression of CRYAB led to reduced colony size in both U87 and LN229 cells, indicating the impediment of glioma cell proliferation (**Figure 9C**). To further validate this observation, the CCK-8 assay was conducted (**Figures 9G, H**).

In order to investigate the impact of CRYAB on glioma cell migration, we utilized both scratch and transwell assays (**Figure 9B**). The outcomes demonstrated that the knockdown of CRYAB significantly impeded the mgration capability of U87 and LN229 cells (**Figures 9D, E**). Consequently, the silencing of CRYAB exhibited inhibitory effects on glioma cell proliferation and migration. Apoptosis is a pivotal process involved in the aggressive characteristics of numerous tumors. To gain deeper insight into the influence of CRYAB on tumor cell apoptosis, we conducted additional investigations. The results from the apoptosis assay demonstrated that downregulation of CRYAB significantly enhanced apoptosis in both U87 and LN229 cell lines (**Figures 9F, I**).

## 4 Discussion

Glioma, the predominant form of primary brain tumor arising from glial cells, represents approximately 80% of all cases of brain tumors (47). The exact etiology of glioma remains unclear, but it has been associated with genetic factors, environmental exposures, and gene mutations (48). The specific mechanisms underlying the origin of glioma are still poorly understood. In clinical practice, the preferred treatment for glioma patients is surgical resection (4). However, the effectiveness of surgery alone is often limited. Additional treatment options include radiation therapy and chemotherapy, although their outcomes are also unsatisfactory (49). Novel approaches such as targeted therapy and immunotherapy have emerged with the aim of interfering with specific signaling pathways in glioma cells or enhancing the immune system to suppress tumor growth. However, the immunosuppressive effects of glioma often contribute to poor treatment responses (50). The expression of CRYAB gene is mainly observed in cardiac and neural tissues, and its dysregulated expression has been linked to the pathogenesis and advancement of diverse immune-associated disorders (51). Research findings indicate that the atypical expression of CRYAB has been implicated in various autoimmune disorders, including rheumatoid arthritis and systemic lupus erythematosus, as well as inflammatory conditions such as pneumonia and myocarditis. The expression of CRYAB may be regulated by inflammatory cytokines and, in turn, can influence the extent and progression of the inflammatory response.In the nervous system, abnormal expression of the CRYAB gene is associated with the occurrence and progression of several neurodegenerative diseases (9). In neurodegenerative conditions, such as Alzheimer’s disease and Parkinson’s disease, aberrant expression of CRYAB has been associated with neuronal apoptosis (cell death) and the progression of neurodegeneration. Moreover, CRYAB has proven to have a significant impact in the context of neurotrauma and inflammatory disorders, including stroke, traumatic brain injury, and spinal cord injury (52).Therefore, the dysregulation of CRYAB expression in cardiac and neural tissues is implicated in the pathogenesis of immune-related diseases, inflammation-related conditions, as well as neurodegenerative disorders. The implication of CRYAB in these pathological conditions highlights its significance as a promising therapeutic target for the development of interventions (8).

In this study, we conducted snRNA-seq analysis on tumor samples from 10 patients with GBM to investigate the major cell types involved in GBM progression. Single-cell sequencing technology has significantly enhanced our exploration of neuroimmune molecular mechanisms, ushering in a redefinition of disease subtyping, and facilitating the discovery of novel therapeutic targets (53, 54). Building upon newly discovered neuroimmune molecular mechanisms and biomarkers, we aim to explore novel therapeutic strategies, including immunotherapy and pharmacological interventions, to enhance treatment outcomes for neurological disorders (55). By utilizing sequencing technologies, we delve more extensively into the regulatory mechanisms of the neuroimmune system, encompassing intercellular interactions, signaling pathways, cytokine release, and regulation, ultimately providing a comprehensive understanding of the processes underlying the onset and progression of glioma. By employing dimensionality reduction clustering techniques, we successfully distinguished seven unique cell populations, namely oligodendroglial cells, neurons, myeloid cells, astrocytes, vascular endothelial cells (VECs), proliferating cells, and T cells. We scored the cells and performed GO-BP enrichment analysis and differential gene analysis. Intracellular heterogeneity was observed within the oligodendroglial cells, which were further divided into four subgroups, including malignant and non-malignant cells. Cell tracking and single-cell trajectory analysis were performed to visualize the differentiation and developmental relationship between oligodendroglial cell and GBM cell subgroups. Using the slingshot method, we further analyzed the trajectory of cell differentiation in GBM and identified two distinct lineages. Cellchat analysis was performed to investigate the signaling communication network among cells and gain insights into their intercellular interactions. Additionally, we investigated the coordinated functions of multiple cell clusters and signaling pathways. Our study revealed that within the terminal differentiation stage of glioma tissue, a subpopulation of oligodendroglial cells exhibited the highest expression of CRYAB, which was associated with prognosis and confirmed by *in vitro* experiments (56, 57). The knockdown of CRYAB in glioma cells resulted in the suppression of cell proliferation and migration, concomitant with the induction of apoptosis.

Immunotherapy is a potent therapeutic strategy in the field of medicine, targeting the immune escape strategies employed by tumors and effectively activating the patient’s immune cells to combat malignant cancer cells (58). The Cryab gene assumes a pivotal role in tumor immunity, as evidenced by the notable distinctions observed in immune infiltration between the high CRYAB+ GBM score cohort and the low CRYAB+ GBM score cohort within the context of this investigation. Indeed, the expression of Cryab is intricately linked to tumor progression and response to treatment. Primarily, there is a frequent upregulation of Cryab within tumor cells (59). The elevated expression of Cryab may be associated with the survival, proliferation, and metastatic potential of tumor cells. It has been observed that increased Cryab levels contribute to enhanced cell viability, proliferation, and the ability of tumor cells to spread to distant sites (60). The overexpression of Cryab in tumor cells may play a vital role in promoting cell survival and offering a means to evade immune system-mediated attacks. Additionally, the presence of Cryab expression has been correlated with tumor-induced immune evasion. The upregulation of Cryab has been observed to be closely linked with the ability of tumors to escape immune detection and subsequent immune responses. The mechanism of immune surveillance involves the recognition and elimination of potential tumor cells by the body’s immune system (61). Nevertheless, tumor cells have the ability to evade immune surveillance through diverse mechanisms. These evasion tactics include downregulating the expression of major histocompatibility complex (MHC) molecules, which are crucial for immune recognition, as well as modulating the expression of immune inhibitory factors (62). Cryab has been found to regulate the immune escape mechanisms of tumor cells, inhibiting the activation of T cells and the expression of tumor-associated antigens, thereby aiding the evasion of immune attacks by tumor cells (63). Tumor cell immune resistance is a major challenge in immunotherapy. Scientific research has revealed a potential correlation between the expression of Cryab and tumor resistance towards immunotherapy. Overexpression of Cryab can render tumor cells insensitive to attacks from immune cells, thereby reducing the effectiveness of immunotherapy (64). In a nutshell, the Cryab gene exerts a pivotal influence on tumor immunity by exerting regulatory control over crucial aspects such as tumor cell survival, immune evasion, and resistance to immune-based therapies.Thus, gaining a comprehensive comprehension of tumor immunology and fostering the advancement of novel immunotherapeutic methodologies hold immense importance.

The association between the CRYAB gene and the prognosis of glioblastoma multiforme (GBM) has been successfully elucidated, alongside an in-depth investigation into its involvement in cellular communication, as well as cellular development and differentiation mechanisms. The findings suggest that increased CRYAB expression is linked to unfavorable prognosis. Several studies have validated a substantial correlation between the presence, advancement, and prognostic implications of cancer and the expression pattern of the CRYAB gene. For example, research has shown that increased levels of CRYAB in glioblastoma patients are closely related to decreased survival rates and increased susceptibility to metastatic diseases. The expression of the CRYAB gene has been identified as a prospective diagnostic indicator for a range of cancers, including prostate cancer, colorectal cancer, and gastric cancer (65). The assessment of CRYAB gene expression levels offers valuable insights into the prognosis and treatment response of various tumors. In particular, CRYAB plays a crucial role in the management of glioblastomas, a type of brain tumor. Research findings indicate that the inhibition of CRYAB expression in glioblastoma cells leads to a reduction in their invasiveness and proliferation rates. Moreover, this silencing of CRYAB expression has been shown to increase the sensitivity of these cells towards chemotherapy drugs (66). The upregulation of CRYAB in glioblastomas suggests its potential role in promoting tumor occurrence and progression, which is consistent with previous research on CRYAB in other cancers. Our study represents the pioneering investigation examining the precise involvement of CRYAB in glioblastomas. The inhibitory effect of CRYAB silencing on glioblastoma cell behavior indicates that targeting CRYAB may be a promising treatment approach. We have validated our research findings through *in vitro* experiments, demonstrating that silencing CRYAB in glioblastoma cells inhibits cell proliferation, migration. In conclusion, our study has established a reliable diagnostic and prognostic model for glioblastoma and provided evidence for the upregulation of CRYAB and its promotion of tumor cell behavior in glioblastomas. Targeting CRYAB may be a promising therapeutic strategy for glioblastoma. Additional investigations are required to gain a comprehensive understanding of the underlying mechanisms through which CRYAB operates in relation to glioblastomas. Moreover, exploring the clinical implications of targeting CRYAB holds promise and warrants further exploration.

## 5 Conclusions

In conclusion, the utilization of CRYAB-related models enables a comprehensive patient classification for prognosis and immunological assessment in glioblastoma patients. Our research findings can provide valuable insights for the detection, treatment, and mechanistic studies of gliomas.

## Data availability statement

The original contributions presented in the study are included in the article/**Supplementary Material**. Further inquiries can be directed to the corresponding authors.

## Ethics statement

Ethical approval was not required for the studies on animals in accordance with the local legislation and institutional requirements because only commercially available established cell lines were used.

## Author contributions

H-BC: Conceptualization, Investigation, Validation, Writing – original draft. M-YZ: Investigation, Writing – original draft. X-HL: Formal Analysis, Writing – original draft. Y-QL: Investigation, Writing – original draft. T-HY: Software, Writing – review & editing. C-ZW: Data curation, Writing – review & editing. L-NW: Methodology, Writing – review & editing. W-YX: Supervision, Writing – review & editing. BL: Formal Analysis, Writing – review & editing. Y-PC: Supervision, Writing – original draft. FZ: Supervision, Validation, Writing – review & editing. W-MH: Supervision, Validation, Visualization, Writing – original draft.

## Supplementary material

Supplementary Figure 1 The classification of GBM cells. According to the inferCNV results, we defined cells with high CNV levels as GBM cells.

Supplementary Figure 2 CRYAB gene transfection knock-down low efficiency verification. Compared with untransfected cells, the mRNA level of CRYAB gene was significantly decreased in the transfected knockdown group.
