# Supplementary figures and images for: Single cell sequencing revealed the mechanism of CRYAB in glioma and its diagnostic and prognostic value
Source: Front Immunol. 2024 Jan 11;14:1336187. doi: 10.3389/fimmu.2023.1336187 (PMC10808695; doi:10.3389/fimmu.2023.1336187)

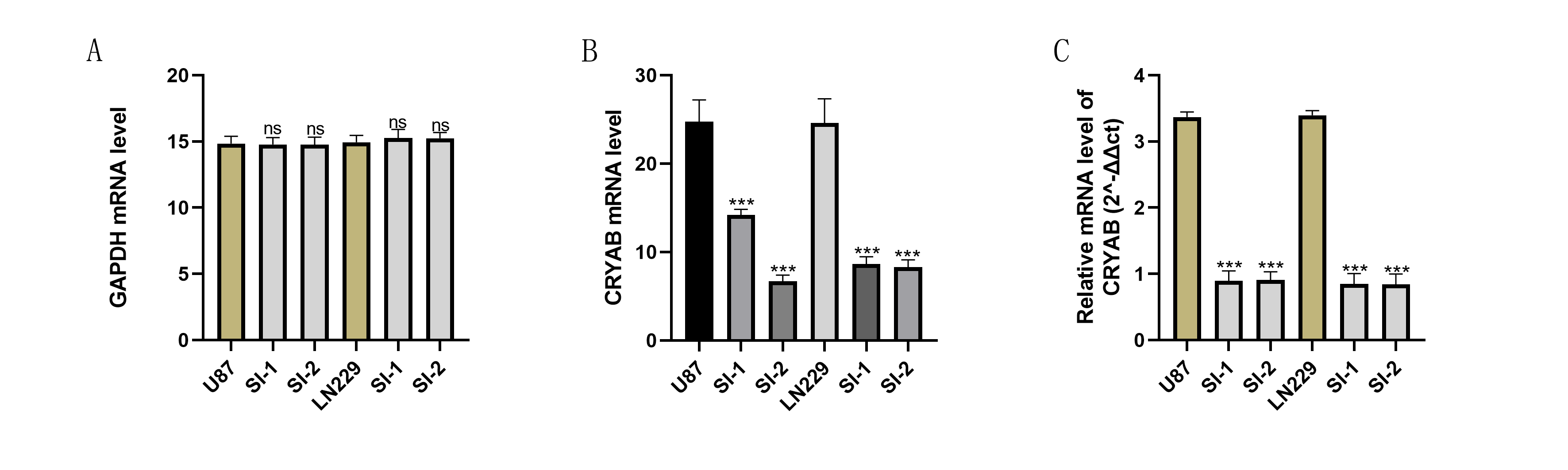

Supplement: Supplementary Figure 2 — CRYAB gene transfection knock-down low efficiency verification. Compared with untransfected cells, the mRNA level of CRYAB gene was significantly decreased in the transfected knockdown group. [file Image_2.tif]
