# Supplementary material for: Single cell sequencing revealed the mechanism of CRYAB in glioma and its diagnostic and prognostic value
Source: Front Immunol. 2024 Jan 11;14:1336187. doi: 10.3389/fimmu.2023.1336187 (PMC10808695; doi:10.3389/fimmu.2023.1336187)
Supplement: Supplementary file 3 [file Table_1.docx]

| **Oligonucleotides** | **Nucleotide sequence (5'-3')** |
| --- | --- |
| **siRNA** |  |
| SiRNA-NC | GCUUCGCGCCGUAGUCUUA |
| Si CRYAB-1 | CCATTACTTCATCCCTGTCAT |
| Si CRYAB-2 | GCAGGCCCAAATTATCAAGCT |
| **Primer** |  |
| GAPDH | GGCCTCCAAGGAGTAAGACC (forward) |
|  | AGGGGAGATTCAGTGTGGTG (reverse) |
| CRYAB | TGCCATACCTTCTCCGAAGC (forward) |
|  | TCCGGGATTTGGCAATGTGA (reverse) |
|  |  |

**Table S1. Oligonucleotides used in research**
